# The earliest molecular response to stretch of insect flight muscle as revealed by fast X-ray diffraction recording

**DOI:** 10.1038/srep42272

**Published:** 2017-02-08

**Authors:** Hiroyuki Iwamoto

**Affiliations:** 1 Japan Synchrotron Radiation Research Institute (JASRI), SPring-8, 1-1-1 Kouto, Sayo-cho, Sayo-gun, Hyogo 679-5198, Japan

## Abstract

Small insects drive their flight muscle at frequencies up to 1,000 Hz. This remarkable ability owes to the mechanism of stretch activation. However, it remains unknown as to what sarcomeric component senses the stretch and triggers the following force generation. Here we show that the earliest structural change after a step stretch is reflected in the blinking of the 111 and 201 reflections, as observed in the fast X-ray diffraction recording from isolated bumblebee flight muscle fibers. The same signal has also been observed in live bumblebee. We demonstrate that (1) the signal responds almost concomitantly to a quick step stretch, (2) the signal grows with increasing calcium levels as the stretch-activated force does, and (3) a full 3-dimensional model demonstrates that the signal is maximized when objects having a 38.7-nm actin periodicity travel by ~20 nm along the filament axis. This is the expected displacement if myosin heads are loosely associated with actin target zones (where actin monomers are favorably oriented), and are dragged by a 1.3% stretch, which effectively causes stretch-induced activation. These results support and strengthen our proposal that the myosin head itself acts as the stretch sensor, after calcium-induced association with actin in a low-force form.

Small winged insects, such as mosquitos and midges, can beat their wings at high frequencies (up to 1,000 Hz)^1^,^2^ (see also ref. 3), and it is difficult to achieve these frequencies by repeating ordinary contraction-relaxation cycles, which require energetically costly calcium release and reuptake by the sarcoplasmic reticulum. Instead, these insects have developed a system of asynchronous flight muscles, in which there is no one-to-one correlation between nerve impulses and wing beats. To date, >500,000 species are known to share the “asynchronous flight muscle” type of physiology, and another trait shared by such flight muscles is the complete “fibrillar” separation between individual non-anastomosing myofibrils of diameter 1–4 microns. This grouping of features, distributed worldwide among 5 different insect orders, is thought to indicate up to 7–10 distinct evolutionary origins^4^.

In asynchronous flight muscle, the contractile machinery is kept activated by a constant level of intracellular calcium, while the two antagonistic flight muscles in the thorax undergo self-sustained oscillations. These self-sustained oscillations are driven by a process called stretch activation (SA), in which an externally applied stretch elicits a delayed rise of contractile force^4^,^5^. Two antagonistic flight muscles alternately pull each other to cause SA, resulting in continuous oscillations. The phenomenon of SA is observed to some extent in vertebrate skeletal and cardiac muscles^6^,^7^, but it is most conspicuous in the asynchronous type of insect flight muscle (IFM). It is clear that the stretch-sensing mechanism is located within the sarcomeric structure, because skinned (demembranated) IFM fiber preparations exhibit SA when stretched under activating conditions. Despite a long history of research, however, it remains controversial as to what element of the sarcomeric structure acts as the primary trigger of SA. It could be the spatial arrangement of myosin and actin monomers in the myofilament lattice^8^ or the IFM-specific isoforms of troponin that has an unusually long extension^9^.

To explore the molecular mechanism of SA in IFM, we have conducted a number of experiments, including fast time-resolved X-ray diffraction recordings on skinned bumblebee IFM fibers with a time resolution of either 3.4 ms^10^ or 0.5 ms^11^. Especially, the latter study started to reveal a previously unnoticed quick X-ray signal that is elicited when a step stretch (complete in 1 ms) is applied to the IFM fiber specimens, namely a transient rise of the 111 reflection and a concomitant diminution of the 201 reflection. These are the earliest changes that occur after a stretch, and they occur ahead of the changes of other reflections that parallel the SA force generation. Therefore, the 111 and 201 reflections are the primary candidates for the reporter of the structural changes that trigger SA.

These signals are even more conspicuous in live bumblebees while they beat their wings, as revealed by fast X-ray diffraction movies (5,000 frames/s)^12^. The movies have shown that the changes of the 111 and 201 reflections occur in the stretch phase of both of the two antagonistic IFMs. From model calculations of 2-dimensional (2-D) filament lattice structure, the paper argues that the reciprocal intensity changes can be explained by a deformation of myosin heads already attached to actin filaments. This has led to a proposal that the SA occurs when the stretch-induced deformation of weakly associated myosin heads causes a force-producing transition.

The purpose of this paper is to give a detailed description of the results of the 0.5-ms time-resolved experiments using skinned IFM fibers and to discuss their implications, as they were only briefly presented at a conference^11^. Experiments using skinned fibers are complementary to those using live insects. Although *ex-vivo* observations may not be identical to what occurs *in vivo*, the skinned fiber preparations allow precise control of experimental conditions, and much smaller background X-ray scattering allows analyses of weaker reflections. In these experiments, X-ray diffraction patterns were taken at 2,000 frames/s instead of 5,000 frames/s for live bumblebees, but the lower experimental temperature (20° vs. ~42° in the thorax of a live bumblebee) gives enough time resolution to record responses to 1-ms step stretches. We further expand the original 2-D model^12^ to a full 3-D model, to explore possible structural changes of contractile proteins in the 3-D space of myofilament lattice. This 3-D model demonstrates that the observed changes of the 111 and 201 reflections are indeed satisfactorily explained, if myosin heads are pulled along the filament axis while they are loosely bound to actin. These results provide further support for the idea that the SA-triggering mechanism is built into myosin itself.

## Results

### Responses of X-ray reflections to stretch of fully calcium-activated skinned IFM fibers from bumblebee

An array of skinned bumblebee IFM fibers were mounted in a specimen chamber, and were step stretched after full calcium activation, and time-resolved X-ray recordings were done at the high-flux BL40XU beamline of SPring-8 as were done previously^10^. The difference was the detector, capable of recording full megapixel (1024 × 1024) frames at 2,000 frames/s. Because of faster recording, we were unable to include both a stretch and a release in a single run of recording, and therefore we focused mainly on the responses to stretch.

**Figure 1:**
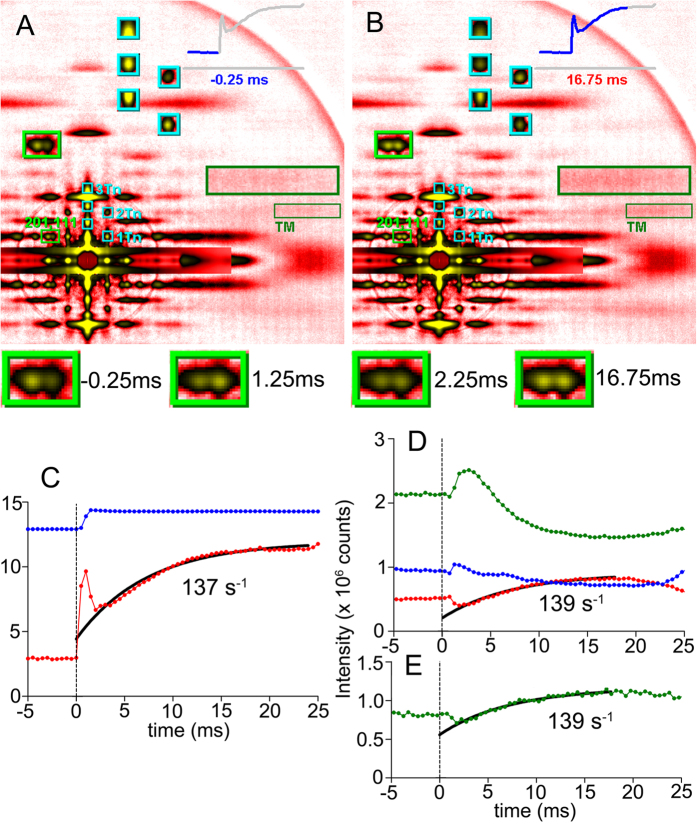
Diffraction movie frames recorded from stretch-activated bumblebee flight muscle fibers at a saturating level of calcium (pH = 4.0) and time courses of changes of force and reflection intensities (time resolution, 0.5 ms). (**A**) and (**B**) Two frames of diffraction movie, taken 0.25 ms before (**A**) and 16.75 ms (**B**) after step stretch. The boxes indicate the reflections of interest, and their magnified views are shown in the upper area of the frame. Cyan boxes, troponin reflections. 1Tn, 2Tn and 3Tn represent the 1st-order off-meridional (101), 2nd-order off-meridional (102) and 3rd-order on-meridional (003) troponin reflections, respectively. Dark green box, 2nd actin layer line (tropomyosin reflection, TM). Light green box, 111 and 201 reflections. This box is further magnified in the four panels below (**A**) and (**B**). (**C**) Force (red) and length (blue), summed for all specimens used. The length step is 1% of fiber length. The force is in arbitrary unit. (**D**) Time course of intensity changes of troponin reflections. Blue, 1st (101); red, 2nd (102); green, 3rd (003). (**E**) 2nd actin layer line (tropomyosin) reflection. The curves were fitted to single exponential association function. The data are sum from 33 sets of parallel-aligned fibers.

Figure 1 shows the time course of intensity changes of major reflections after a stretch, along with two X-ray diffraction frames taken immediately before and after a stretch. The reciprocal intensity changes of 101 and 102 and the rise of the 2nd actin layer line reflection (ALL) parallel the rise of stretch-activated (SA) force, as reported previously (Iwamoto *et al*.^10^). The former two reflections originate from the helical arrangement of troponin complexes on 6 actin filaments surrounding a single myosin filament^13^. Their reciprocal intensity changes occur when myosin strongly binds to the actin target zone (where actin monomers are favorably oriented for interaction with myosin), which is located midway between two neighboring troponin complexes on an actin filament^13^. The 2nd ALL reports the movement of tropomyosin on the actin filament, as it is calcium-activated^14^,^15^. It is enhanced when tropomyosin moves to its activating position. The improved quality of diffraction patterns allowed us to determine the time constants for intensity changes. They all have similar values of ~137 s^−1^, which are close to that for the rise of SA force. Unlike in the IFM from giant waterbug^16^, there is no sign that the rise of the 2nd ALL (tropomyosin movement) precedes strong myosin attachment and SA force production. The movies of diffraction patterns are provided as Supplemental Materials.

**Figure 2:**
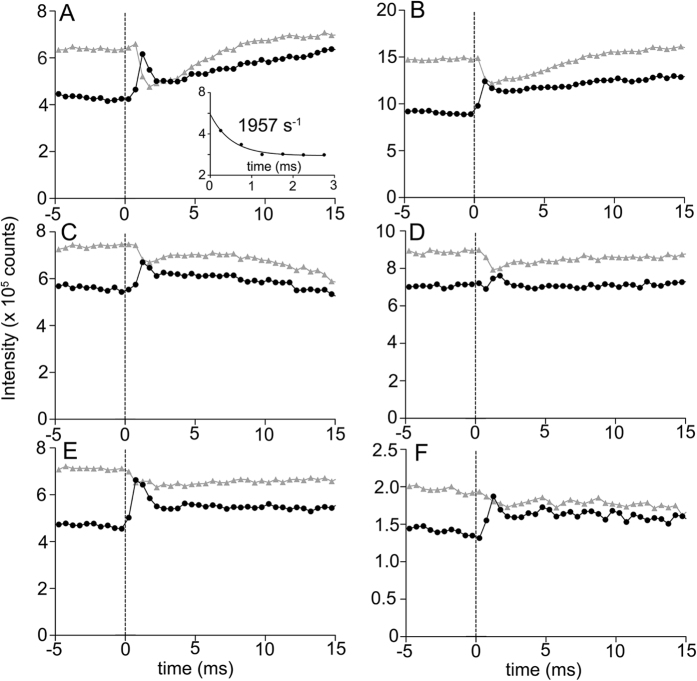
Responses of the 111 and 201 reflections to a step stretch under various conditions. (**A**) pCa = 4.0; (**B**) pCa = 5.75; (**C**) pCa = 6.25; (**D**) relaxed; (**E**) pCa = 4.0, in the presence of 100 μM blebbistatin; (**F**) Igase-treated fibers, pCa = 4.0, in the presence of 100 μM blebbistatin. Black, 111; gray, 201. The inset in (**A**) is the fitting of the fast decay of 111 to a single exponential decay function. The numbers of fiber sets used: (**A**) 33; (**B**) 27; (**C**) 19; (**D**) 27; (**E**) 19; (**F**) 10.

As shown in the magnified parts of the diffraction patterns in Fig. 1, reciprocal intensity changes of the 111 and 201 reflections are observed after a stretch. Before the stretch, the 111 is weaker than 201, but this is reversed immediately after the stretch. Their time courses are shown in Fig. 2A. As shown, the 111 intensity rises almost concomitantly with the 1-ms stretch, but the rise is transient. It falls quickly with a rate constant of ~1,500 s^−1^, and then starts to rise again slowly with a time course similar to those of other reflections such as 102. The 201 reflection falls at the moment of stretch, and after that it starts to rise slowly as 111 does, and therefore the changes of the 111 and 201 are no longer reciprocal.

### Factors that affect the magnitude of changes of the 111 and 201 reflections

Skinned bumblebee IFM specimens exhibit greater magnitude of SA force at higher concentrations of calcium. To determine the calcium dependence of the responses of 111 and 201 reflections, we recorded diffraction movies at varied concentrations of calcium. As shown in Fig. 2A–D, the responses of the 111 and 201 to stretch are diminished as calcium is lowered, and relaxed IFM fibers show only small responses to stretch. The magnitude of intensity changes is not affected by the presence of blebbistatin^17^, a myosin inhibitor that practically eliminates calcium- and stretch-activated forces (Fig. 2E). In the presence of blebbistatin, the subsequent slow rise of 111 as observed in control (Fig. 2A) is absent. The rise of the 111 (in the presence of blebbistatin) remains intact in fibers treated with Igase (Fig. 2F), an endoproteinase that specifically cleaves a sequence found in the long C-terminal extension of troponin-I^18^. The enzyme has been shown to almost completely (97%) remove the extension in bumblebee IFM, while leaving IFM’s functions intact^19^.

### Effect of step release on reflection intensities

**Figure 3:**
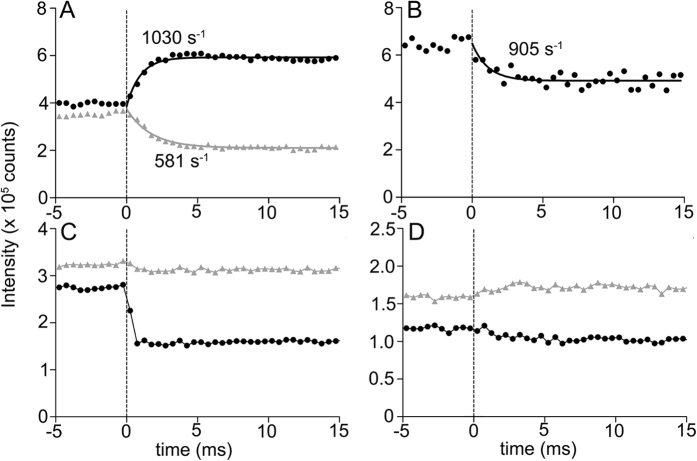
Responses of reflections to a step release of bumblebee flight muscle fibers activated at pCa = 4.0. (**A**) The responses of the 1st (black) and 2nd (gray) troponin reflections (101 and 102). (**B**) The response of the 2nd actin layer line (tropomyosin) reflection. (**C**) The responses of the 111 (black) and 201 (gray) reflections. (**D**) The responses of the 111 (black) and 201 (gray) reflections but in the presence of 100 uM blebbistatin. The curves in (**A**) and (**B**) were fitted to a single exponential decay or association function. The number of fiber sets used: 19.

So far we have focused on the effect of step stretch to the 111 and 201 reflections, but it is also of interest to perform a reverse type of experiment, i.e., applying a step release to IFM fibers that are already stretch-activated by a preceding step stretch. Figure 3 shows the responses of some reflections to a 1-ms step release of fully calcium-activated bumblebee IFM fibers. To confirm previous results, 102 troponin reflection falls and the 101 rises after release (Fig. 3A), indicating that the strongly bound myosin heads are leaving the actin target zone. Although their changes are much faster than after stretch, we were able to determine their rate constants owing to improved time resolution. The rate constants are in the range of 500–1,000 s^−1^, and are much greater than those after a step stretch. The 2nd actin layer line also falls in an exponential manner, and its rate constant is comparable to those for 101 and 102 (Fig. 3B).

The step release has striking effects on the 111 and 201 reflections. The release causes a sharp and immediate fall of the 111 reflection (Fig. 3B). Unlike in the case of stretch, the response is not transient, and the 111 stays constant at the lowest level for at least 15 ms (Fig. 3C). On the contrary, the 201 is not affected by the release. Thus, the changes of the 111 and 201 are not reciprocal. In the presence of blebbistatin, the sharp fall of the 111 is greatly diminished, but the two reflections seem to change in a reciprocal manner (Fig. 3D). As will be discussed, this is expected because blebbistatin keeps myosin heads in the weakly binding form.

### Responses of 111 and 201 in other insect species

To determine whether the observed behavior of the 111 and 201 reflections is specific to bumblebee or common among other insect species, we recorded time-resolved diffraction patterns from IFM fibers of a giant waterbug (*Lethocerus deyrollei*, Hemiptera), and a giant crane fly (*Ctenacroscelis mikado*, Diptera). Under relaxing conditions, the responses of the 111 and 201 reflections are variable, and in giant waterbug, the 111 drops upon stretch. At a saturating calcium concentration, however, IFM fibers from both species exhibited quick and transient reciprocal changes of 111 and 201, like in bumblebee fibers (Suppl. Fig. 1). Therefore, it is likely that the observations of the 111 and 201 reflections made for bumblebee IFM are shared by other insect species.

## Discussion

In this paper we described the response of X-ray reflections to a step stretch of isolated and skinned bumblebee IFM fibers at a sub-millisecond time resolution, with a special emphasis on the behavior of the 111 and 201 reflections. The major findings are:

- Reciprocal intensity changes of the 111 and 201 reflections occur almost concomitantly with the 1-ms stretch. These intensity changes are followed by a rapid decay, indicating that the structures causing these intensity changes undergo rapid stress relaxation.
- The intensity changes of the 111 and 201 are calcium dependent, and occur only weakly in the relaxed state. They are not affected by the presence of blebbistatin, an inhibitor that blocks strong actin-myosin interaction.
- The intensity changes of the 111 and 201 are not affected by the enzymatic removal of the long C-terminal extension of IFM troponin-I. This precludes the possibility that the extension contributes to the intensity changes.
- The initial transient changes of 111 and 201 intensities are followed by slower changes (not reciprocal but parallel rise) with time courses similar to those of reflections reporting strong myosin binding.
- Upon step release of stretch-activated fibers, the 111 drops quickly and then remains at a constant level for a long period. The 201 is insensitive to the release. Thus, the process that occurs upon release is not a simple reversal of the response to stretch.
- The responses of the 111 and 201 to stretch are also observed in other insect species that have asynchronous IFM (giant waterbug and giant crane fly).

As it is clear from these observations, the changes of the 111 and 201 reflections are reciprocal at the time of stretch, but this is not always true under other circumstances. Thus these reflections seem to be influenced by multiple factors. In the following, we will discuss what factors can influence these reflections by using 3-D models.

The 111 and 201 reflections are indexed to a crystal lattice with the unit cell size of 38.7 nm in the axial direction. Therefore, if their intensities change upon stretch or release of IFM fibers, it is inferred that the length change shifts the position of some object relative to 38.7 nm-spaced structures (actin target zones and troponin complexes), and alters the interference between them. These reflections are also a part of the 1st actin and 3rd myosin layer line reflections. Because the 1st actin layer line reflection is known to be enhanced when myosin heads are strongly bound to actin, it is expected that these reflections will be enhanced upon formation of strongly bound heads, as well as other reflections on the same layer line (e.g., 211 and 311). Thus the slow parallel rise of the 111 and 201 following their initial transient changes (a) is likely to reflect this process. On the other hand, myosin layer line reflections tend to weaken in activated fibers as myosin heads leave the regular helical array of myosin filaments. Therefore it is unlikely that the strong enhancement of the 111 as observed in live bumblebee^12^ is caused by a structural change of the myosin filament.

**Figure 4:**
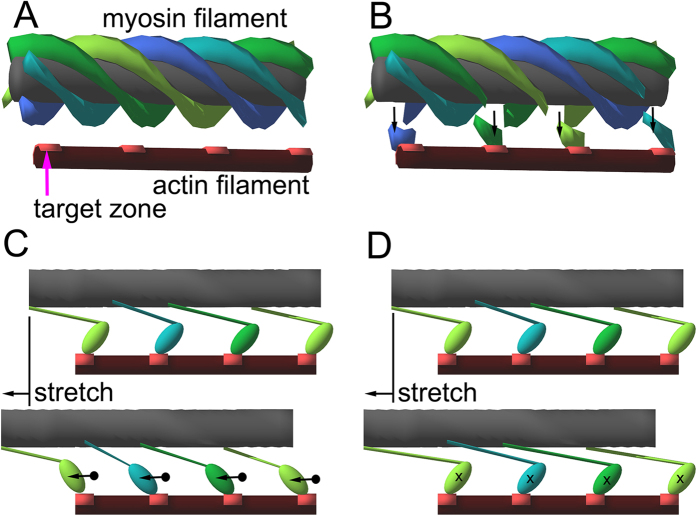
Schematic drawings of the ideas underlying the model calculations. These drawings summarize structures that can move upon stretch relative to the troponin complexes, causing the change in X-ray interference and resulting in the intensity change of reflections. (**A**) In the relaxed state. The 4-start helical array of myosin heads (drawn as continuous helical strands) is the only structure that can move with respect to the troponin complexes (located midway between the neighboring actin target zones, not drawn in the figure). (**B**) Calcium-activated state. The myosin heads close to the target zone leave the helical array (arrows), creating defects in the helical strands. The periodicity of the defects is the same as that of the target zones (or troponin complexes, 38.7 nm) so that these can be sources of interference. (**C**) Movement of actin-attached myosin heads upon stretch in the weakly binding state. Because of the limited interaction in the actin-myosin interface, a large movement of the center of mass of the myosin head is expected (dots and arrows). (**D**) Movement of actin-attached myosin heads upon stretch in the strongly binding state. Because of the extensive interaction in the interface, the movement of the center of mass is limited. In (**C** and **D**) the myosin heads not bound to actin (helical arrays with defects) are not drawn.

The question, then, is what object moves relative to the array of 38.7 nm-spaced structures. Here, before starting to describe the details of the model calculations, it would be useful to illustrate the basic idea behind the model calculations, as in Fig. 4.

In the relaxed state, we assume that all the myosin heads form a regular 4-start helix on the myosin filament backbone (Fig. 4A). Upon calcium activation, myosin heads close to the target zones on the actin filaments (bright-red area on the actin filament in Fig. 4) are expected to attach to the actin filaments. This disrupts the helical strands of myosin heads, creating defects (voids) on the myosin helix (Fig. 4B). The defects occur with a periodicity of the target zones (38.7 nm), and can interfere with the troponin complexes in terms of X-ray diffraction.

Myosin heads that have left the myosin helix may attach to actin either in a weakly-binding form (Fig. 4C) or in a strongly-binding form (Fig. 4D). The weak bond may be formed electrostatically in the limited regions of the myosin and actin molecules, e.g., in the lysine-rich flexible loop between the 50 k–20 k junction of the myosin heads and the acidic N-terminus of actin^20^. Because of this limited nature of actin-myosin interaction, both the motor domain and the light chain domain of the myosin head are expected to be mobile, and when the muscle fiber is stretched, the center of mass of the head is expected to move substantially along the filament axis (dot and arrow in Fig. 4C).

On the other hand, in the strongly-bound actomyosin complex, more extensive interaction is involved in the interface, including hydrophobic interaction. This interaction would limit the mobility of the myosin head, resulting in a much smaller movement of the center of mass upon stretch of the muscle fiber (cross in Fig. 4D).

To summarize this section, the structures that move with respect to the troponin complex upon stretch (possible source of X-ray intensity change upon stretch) are as follows:

- In the relaxed state, the helical array of myosin heads (without defect).
- In the weakly binding state, a major part of actin-bound myosin head and the helical array of myosin heads with defect.
- In the strongly binding state, the helical array of myosin heads with defect, and can involve a small part of actin-bound myosin head (possibly the light-chain domain).

Having described the fundamental idea behind the model calculations, we will look into the result of calculations in each case.

**Figure 5:**
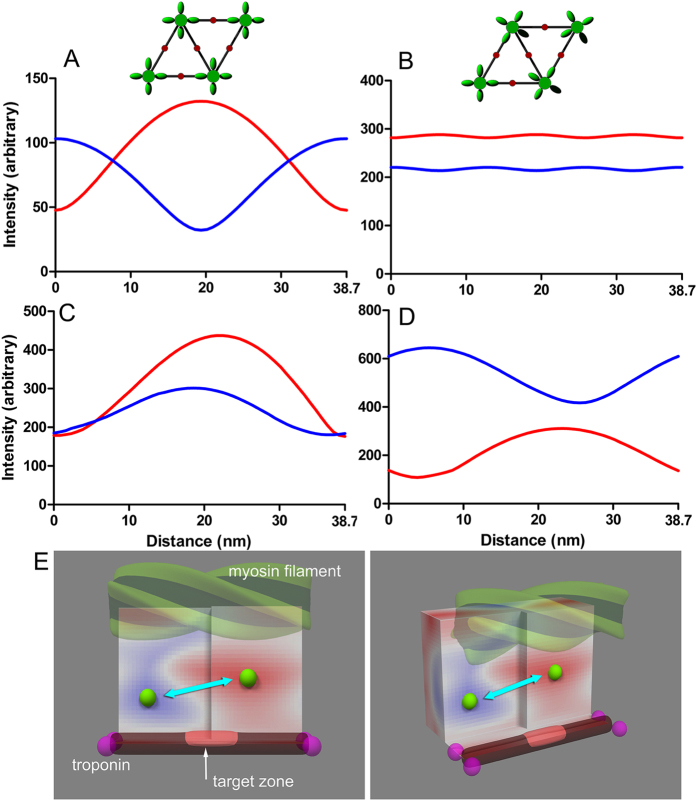
Model calculations of intensity changes of the 111 and 201 reflections upon stretch of IFM fibers. (**A** and **B**) Effect of sliding of the myosin filament (helical object in Fig. 4A) past the actin filament (thinner rod below the myosin filament in Fig. 4A). This corresponds to the stretch of a relaxed IFM fiber. Red curve, 111; blue curve, 201. In (**A**) The rotational angles of the myosin filaments are in register. In (**B**) The myosin filaments axially staggered by 38.7/3 nm with respect to the neighboring filaments. (**C** and **D**) Calculations simulating the calcium-activated state in which strongly bound state of myosin has not been formed. In (**C**) The myosin heads nearest to the target zones are attracted to the actin filaments, but they are so disordered that they are invisible by X-ray. Because of this, the myosin helix has defects (see Fig. 4B). The stagger of myosin filaments is as in (**B**). The graph in (**D**) is the combination of (**C**) and (**E**) In which myosin filaments with defects slide past actin filament by 38.7 nm, while the weakly bound myosin heads travel between the hot spot and the cool spot in (**E**). (**E**) Schematic 3-dimensional map for the relative intensities of the 111 and 201 reflections when a mass is located in the 3-dimensional space of the unit cell. This calculation is to predict what happens to the 111 and 201 reflection intensities when weakly bound heads are moved by a stretch as in Fig. 4C. In the red area, the 111 is more intense than 201 and in the blue area, vice versa. The space is cut along the plane containing the spot that gives the highest 111 over 201 (hot spot, the most reddish part) and that containing the spot that gives the lowest 111 with respect to 201 (cool spot, the most bluish part). The greatest reciprocal intensity changes will occur if the mass travels between these two spots (light blue arrow). The magenta balls represent troponin complexes.

In relaxed fibers, the only thing that can move past the troponin array would be the helical array of myosin heads. Although the helical pitch of the myosin filament is different from that of the actin filament, they have a relatively small least common multiple (116 nm)^13^. Therefore, the density distribution along the myosin filament is sufficiently “bumpy”, when viewed from the troponin array, to cause length-dependent variations in the extent of interference. The model calculation shows that, when the myosin filaments are all in register (both axially and rotationally), the sliding of the myosin helix relative to the troponin array causes substantial intensity changes of 111 and 201 (Fig. 5A).

On the contrary, if the myosin filament are not in register^21^, the intensity changes are greatly diminished, meaning a flattened probability of interaction between myosin heads and actin target zones (Fig. 5B; the neighboring myosin filaments are axially staggered by a third of 38.7-nm periodicity). The present X-ray diffraction results for relaxed fibers show that the 111 and 201 reflections change little (Fig. 2), indicating that the myosin filaments in bumblebee IFM are not in register, meaning either systematically rotated, staggered or disordered.

In other insect species, the behavior of the 111 and 201 reflections under relaxing conditions is variable. Especially, the 111 drops upon stretch of giant waterbug fibers (Supplementary Fig. 1b). Possibly the spatial relationship of the myosin and actin filaments in the relaxed state may be variable among different species, but their behavior becomes unanimously reciprocal after calcium-activation (Fig. 2, Supplementary Fig. 1a,c). In other words, regardless of the extent of myosin-filament register in the relaxed state, the myosin heads closest to the actin target zone are recruited upon calcium activation, and thereafter, the movements of these heads with respect to the troponin complexes (and the resulting intensity changes) are more or less the same regardless of the insect species. This may explain why, in general, the IFMs from species other than the giant waterbug have poorer myofilament register (as is evident from their diffraction patterns), and seems unfavorable for the “match-mismatch mechanism”^8^ to operate, but they are equally stretch-activatable.

The motor nerve impulses cause an elevation of the intracellular calcium level, leading to exposure of actin target zones, the number of which depending on the calcium concentration. The myosin heads that can reach the target zone will leave the filament backbone, creating defects in the myosin helix. On average, these defects have a 38.7-nm periodicity, and are expected to cause interference with the troponin array. In fact, the sliding of myosin helices with such defects starts to cause greater intensity changes of the 111 and 201, even if the helix orientations are not in register (Fig. 5C). In this case the intensity changes are not reciprocal.

Next we consider the effect of the myosin heads that are associated with the target zones. If they are immediately bound strongly to actin, they will not move when an externally applied stretch makes the myosin filament slide past the troponin array (because the main body of the myosin heads are stereospecifically affixed on the target zones; see Fig. 4D), and therefore cause no intensity changes. If they are weakly bound, e.g., via the flexible loop 2 of the motor domain^22^, the main body of the myosin head will still move with the myosin filaments as they slide past the actin filaments (see Fig. 4C). Figure 5E shows the effect of myosin head movement in the 3-D space of the unit cell (here the effect of the movement of the myosin helix is not considered). This is in reality the full 3-D version of the 2-D calculations reported previously^12^. Like in the previous report, there is a hot spot (red) that gives the highest 111/201 ratio when the myosin head is located there, and a cool spot (blue) that gives the lowest 111/201 ratio, and their reciprocal intensity changes will be maximized when the myosin head travels between the two spots. They are located across an actin target zone and are separated by ~20 nm along the filament axis, or 1.3% of the half sarcomere length. This is the amount of stretch that effectively causes SA. Thus, the large reciprocal intensity changes as observed in live insects^12^ are expected if the myosin filament backbone pulls the main part of the myosin head from the cool spot to the hot spot, while remaining associated with the target zone. A part of myosin head (possibly the loop 2) will be extended in the process, and this gives a plausible candidate for the trigger to initiate weak- to strong conversion. The viscoelastic recoil of the loop may partly pull back the main part of the myosin head, causing the 111/201 changes to relax. It is a kinetic step following this weak bond formation (entry to the strong binding state) that is inhibited by blebbistatin^23^. To support this, the transient response of the 111/201 is not followed by slower parallel rises in the presence of blebbistatin (Fig. 2E).

In the discussion stated above, the sliding of myosin filaments (with defects) was not considered, but when the real IFM fibers are stretched, both filament sliding and the myosin head motion are expected to occur. When the sliding of myosin filaments with defects (Fig. 5C) is combined with the calculation in Fig. 5E, the intensity changes of the 111 and 201 are clearly reciprocal (Fig. 5D). Thus, the movement of the weak binding heads is considered to be the primary factor to determine the reciprocal intensity changes of the 111 and 201.

Finally, we consider the process that occurs when a step release is applied to fibers that are already stretch-activated. The results show that the 111 reflection shows a concomitant decrease with the release, and stays at the decreased level, while the 201 remains unchanged. This kind of behavior is expected because all that moves with respect to the troponin array are the myosin filaments with defects, and the closest analogy is found in the situation shown in Fig. 5C (non-reciprocal intensity changes of 111/201). The myosin heads are strongly bound to actin, so that they are not expected to move upon release (see Fig. 4D), except for some small elastic recoil of the light chain-binding domain.

To summarize the discussion so far, the present findings for skinned bumblebee IFM fibers and the results for live bees^12^ are best explained if, after calcium activation, myosin heads close to the actin target zones become weakly associated with them and, upon stretch, they travel between two areas across the target zone which give low and high 111/201 ratios, respectively. In the process of the travel, the myosin site for weak interaction with actin, such as the flexible loop 2, may be pulled to initiate the process of SA. These weakly associated myosin heads may be identical to the pre-force cross-bridges described for cryo-fixed giant waterbug fibers^24^,^25^.

On the other hand, an alternative mechanism for SA has been proposed. In these studies, a number of myosin cross-bridges are found at the positions of troponin complexes in relaxed IFM fibers from giant waterbug, *Lethocerus*^24^,^25^. It is postulated that these “troponin bridges” may directly pull tropomyosin and lift its steric blocking, inducing SA. Then a question arises whether the myosin heads discussed here are the “troponin bridges” bound at the position of troponin rather than at the target zone. Actually the 3-D map in Fig. 5E is a repeating unit and it is also true that there are a hot spot and a cool spot on the opposite sides of a troponin complex. Therefore the present results are not incompatible with the idea of “troponin bridges”. However, the troponin bridges differ from the weak-binding (pre-SA) myosin heads as discussed here in that they already exist in relaxed fibers. Our data show that also in *Lethocerus*, the reciprocal intensity changes of 111 and 201 are calcium dependent (Supplemental Material). Therefore, the weak binding heads as discussed here are considered distinct from the “troponin bridges”. If the troponin bridges initiate SA by moving tropomyosin, its movement as monitored by the intensity of the 2nd actin layer line should precede the development of SA force or the changes of 101 or 102 reflections, and in *Lethocerus*, the results supports this^16^. We have also shown that the in bumblebee IFM, tropomyosin can move very rapidly^10^ and the present study indicates that after a step release, its rate constant of movement can reach 500~800 s^−1^. After a step stretch, however, its rate constant is not different from those of SA force and the 201 reflection. Therefore, at least in bumblebee, there is no evidence that the tropomyosin movement precedes SA, and the concomitant rise of the 2nd actin layer line and the SA force may represent the cooperative activation process of the thin filament regulatory system well documented for vertebrate skeletal muscle preparations^26^. The observed difference of the tropomyosin behavior between the giant waterbug and the bee may be because the two species are distantly related, or due to the difference in experimental protocols (step stretch vs. sinusoidal oscillation).

To conclude, detailed analysis of the intensity changes of the 111 and 201 reflections upon stretch of bumblebee IFM, and the full 3-D model calculations as presented here suggest that the most likely explanation for the conspicuous changes of these reflections is that they are caused by low force myosin heads that are formed in a calcium-dependent manner. They are presumably associated with the actin target zone but remain flexible so that their masses are dragged along the filament axis as the fibers are pulled. This gives a most straight forward mechanism of SA in which the drag-induced deformation of these heads triggers a force-generating transition, in support of our earlier proposal from the study using live insects^12^. It is worth repeating the implications of this proposal that IFM utilizes a mechanism of stretch-induced force augmentation shared by vertebrate skeletal^27^ and probably cardiac muscles, and therefore the study of IFM is expected to serve for better understanding of our own muscles.

## Materials and Methods

Skinned IFM fibers from bumblebee (*Bombus ignitus*), giant crane fly (*Ctenacroscelis mikado*), or giant water bug (*Lethocerus deyrollei*) were prepared, stored, mounted and activated as described previously^10^,^28^. In short, single skinned IFM fibers from bumblebee or crane fly were split along the fiber axis to make 2–3 strips, and 7 strips were parallel-aligned as a set by using a pair of ceramic chips. In the case of giant waterbug, a bundle of ~3 single fibers was used instead of a single strip from bumblebee or crane fly. Time-resolved X-ray diffraction studies were performed at the high-flux BL40XU beamline of SPring-8^29^, and the protocol was basically the same as in the previous experiments^10^ except for the use of fast CMOS video camera (SA1.1 or SA5, Photron Inc, Japan) at a frame rate of 2,000/s. A step stretch or release (1% fiber length, complete in 1 ms) was applied during exposure while the fibers were continually moved along the fiber axis at a speed of 100 mm/s to reduce radiation damage^30^,^31^. The diffraction patterns from different fiber sets were summed and reflection intensities were measured after subtraction of background scattering^10^,^32^.

To inhibit myosin activity, blebbistatin (Calbiochem) was added at a concentration of 100 μM. To remove the C-terminal extension of troponin-I (troponin-H), the fibers were treated with Igase (Mobitec, Germany) overnight as described^19^.

The 3-D model calculations were done by using a model filament lattice structure, which has published helical symmetries of actin, myosin and troponin^13^. Myosin heads and troponin complexes were represented as dots, and their form factors were not considered. The myosin filament backbone, actin monomers and tropomyosin were regarded to form continuous rods, and were therefore not included in calculation. The intensities of the 111 and 201 reflections were calculated by vectorially summing the contributions of all the components in the unit cell, after determining their phases with respect to the corresponding lattice planes.

## Additional Information

**How to cite this article:** Iwamoto, H. The earliest molecular response to stretch of insect flight muscle as revealed by fast X-ray diffraction recording. *Sci. Rep.* **7**, 42272; doi: 10.1038/srep42272 (2017).

**Publisher's note:** Springer Nature remains neutral with regard to jurisdictional claims in published maps and institutional affiliations.

## Supplementary Material

Supplementary Document

Supplementary Movie 1

Supplementary Movie 2

Supplementary Movie 3

Supplementary Movie 4

Supplementary Movie 5

## References

[b1] Sotavalta O. The flight-tone (wing-stroke frequency) of insects. Acta Ent. Fenn. 4, 1–117 (1947).

[b2] Sotavalta O. Recordings of high wing-stroke and thoracic vibration frequency in some midges. Biol. Bull. 104, 439–444 (1953).

[b3] Iwamoto H. X-ray diffraction pattern from the flight muscle of *Toxorhynchites towadensis* reveals mosquito’s specific phylogenic position in Diptera. Zool. Lett. 1, 24 (2015). 10.1186/s40851-015-0024-1 PMC4657346 26605069

[b4] Josephson R. K., Malamud J. G. & Stokes D. R. Asynchronous muscle: a primer. J. Exp. Biol. 203, 2713–2722 (2000). 10952872 10.1242/jeb.203.18.2713

[b5] Pringle J. W. S. The Croonean lecture, 1977: Stretch activation of muscle: function and mechanism. Proc. R. Soc. Lond. B 201, 107–130 (1978). 27795 10.1098/rspb.1978.0035

[b6] Kawai M. & Brandt P. W. Sinusoidal analysis: a high-resolution method for correlating biochemical reactions with physiological processes in activated skeletal muscles of rabbit, frog and crayfish. J. Muscle Res. Cell Motility 1, 279–303 (1980). 10.1007/BF00711932 6971874

[b7] Vemuri R. . The stretch-activation response may be critical to the proper functioning of the mammalian heart. Proc. Natl. Acad. Sci. USA 96, 1048–1053 (1999). 9927691 10.1073/pnas.96.3.1048 PMC15348

[b8] Wray J. S. Filament geometry and the activation of insect flight muscles. Nature, 280, 325–326 (1979).

[b9] Bullard B. & Pastore A. Regulating the contraction of insect flight muscle. J. Muscle. Res. Cell Motil. 32, 303–313 (2011). 22105701 10.1007/s10974-011-9278-1

[b10] Iwamoto H., Inoue K. & Yagi N. Fast X-ray recordings reveal dynamic action of contractile and regulatory proteins in stretch-activated insect flight muscle. Biophys. J. 99, 184–192 (2010). 20655846 10.1016/j.bpj.2010.04.009 PMC2895363

[b11] Iwamoto H. & Yagi N. Sub-millisecond time-resolved 2-dimensional X-ray diffraction recording from stretch-activated flight muscle from bumblebee. Biophys. J. 100, 11a–12a (2011).21190652

[b12] Iwamoto H. & Yagi N. The molecular trigger for high-speed wing beats in a bee. Science 341, 1243–1246 (2013). 23970560 10.1126/science.1237266

[b13] Tregear R. T. . X-ray diffraction indicates that active cross-bridges bind to actin target zones in insect flight muscle. Biophys. J. 74, 1439–1451 (1998). 9512040 10.1016/S0006-3495(98)77856-7 PMC1299490

[b14] Huxley H. E. Structural changes in the actin- and myosin-containing filaments during contraction. Cold Spring Harbor Symp. Quant. Biol. 37, 361–376 (1973).

[b15] Parry D. A. D. & Squire J. M. Structural role of tropomyosin in muscle regulation: Analysis of the X-ray diffraction patterns from relaxed and contracting muscles. J. Mol. Biol. 75, 33–55 (1973). 4713300 10.1016/0022-2836(73)90527-5

[b16] Perz-Edwards R. J. . X-ray diffraction evidence for myosin-troponin connections and tropomyosin movement during stretch activation of insect flight muscle. Proc. Natl. Acad. Sci. USA 108, 120–125 (2011). 21148419 10.1073/pnas.1014599107 PMC3017141

[b17] Straight A. F. . Dissecting temporal and spatial control of cytokinesis with a myosin II inhibitor. Science 299, 1743–1747 (2003). 12637748 10.1126/science.1081412

[b18] Clayton J. D., Cripps R. M., Sparrow J. C. & Bullard B. Interaction of troponin-H and glutathione S-transferase-2 in the indirect flight muscles of *Drosophila melanogaster*. J. Muscle Res. Cell Motil. 19, 117–127 (1998). 9536439 10.1023/a:1005304527563

[b19] Iwamoto H. The long C-terminal extension of insect flight muscle-specific troponin-I isoform is not required for stretch activation. Biochem. Biophys. Res. Commun. 431, 47–51 (2013). 23291173 10.1016/j.bbrc.2012.12.101

[b20] Kabsch W., Mannherz H. G., Suck D., Pai E. F. & Holmes K. C. Atomic structure of the actin:DNase I complex. Nature. 347, 37–44 (1990). 2395459 10.1038/347037a0

[b21] Squire J. M. Muscle filament lattices and stretch-activation: the match-mismatch model reassessed. J. Muscle Res. Cell Motil. 13, 183–189 (1992). 1597512 10.1007/BF01874155

[b22] Rayment I. . Three-dimensional structure of myosin subfragment-1: A molecular motor. Science 261, 50–58 (1993). 8316857 10.1126/science.8316857

[b23] Ramamurthy B., Yengo C. M., Straight A. F., Mitchison T. J. & Sweeney H. L. Kinetic mechanism of blebbistatin inhibition of nonmuscle myosin IIb. Biochemistry 43, 14832–14839 (2004). 15544354 10.1021/bi0490284

[b24] Wu S. . Electron tomography of cryofixed, isometrically contracting insect flight muscle reveals novel actin-myosin interactions. PLoS One, 5, e12643 (2010). 20844746 10.1371/journal.pone.0012643 PMC2936580

[b25] Wu S. . Structural changes in isometrically contracting insect flight muscle trapped following a mechanical perturbation. PLoS One 7, e39422 (2012). 22761792 10.1371/journal.pone.0039422 PMC3382574

[b26] Lehrer S. S. & Morris E. P. Dual effects of tropomyosin and troponin-tropomyosin on actomyosin subfragment-1 ATPase. J. Biol. Chem. 257, 8073–8080 (1982). 6123507

[b27] Iwamoto H. Strain sensitivity and turnover rate of low force cross-bridges in contracting skeletal muscle fibers in the presence of phosphate. Biophys. J. 68, 243–250 (1995). 7711247 10.1016/S0006-3495(95)80180-3 PMC1281682

[b28] Iwamoto H. Evidence for unique structural change of the thin filaments upon calcium-activation of insect flight muscle. J. Mol. Biol. 390, 99–111 (2009). 19433094 10.1016/j.jmb.2009.05.002

[b29] Inoue K. . Present status of high flux beamline (BL40XU) at SPring-8. Nucl. Instr. Methods Phys. Res. A 467/8, 674–677 (2001).

[b30] Wakayama J., Tamura T., Yagi N. & Iwamoto H. Structural transients of contractile proteins upon sudden ATP liberation in skeletal muscle fibers. Biophys. J. 87, 430–441 (2004). 15240477 10.1529/biophysj.103.035063 PMC1304364

[b31] Tamura T., Wakayama J., Inoue K., Yagi N. & Iwamoto H. Dynamics of thin filament activation in rabbit skeletal muscle fibers examined by time-resolved X-ray diffraction. Biophys. J. 96, 1045–1055 (2009). 19186142 10.1016/j.bpj.2008.09.022 PMC2716644

[b32] Iwamoto H., Wakayama J., Fujisawa T. & Yagi N. Static and dynamic X-ray diffraction recordings from living mammalian and amphibian skeletal muscles. Biophys. J. 85, 2492–2506 (2003). 14507712 10.1016/s0006-3495(03)74672-4 PMC1303473

