# Supplementary material for: The earliest molecular response to stretch of insect flight muscle as revealed by fast X-ray diffraction recording
Source: Sci Rep. 2017 Feb 8;7:42272. doi: 10.1038/srep42272 (PMC5296744; doi:10.1038/srep42272)
Supplement: Supplementary Document [file srep42272-s1.doc]

Supplementary document

**The earliest molecular response to stretch of insect flight muscle as revealed by fast X-ray diffraction recording**

Hiroyuki Iwamoto


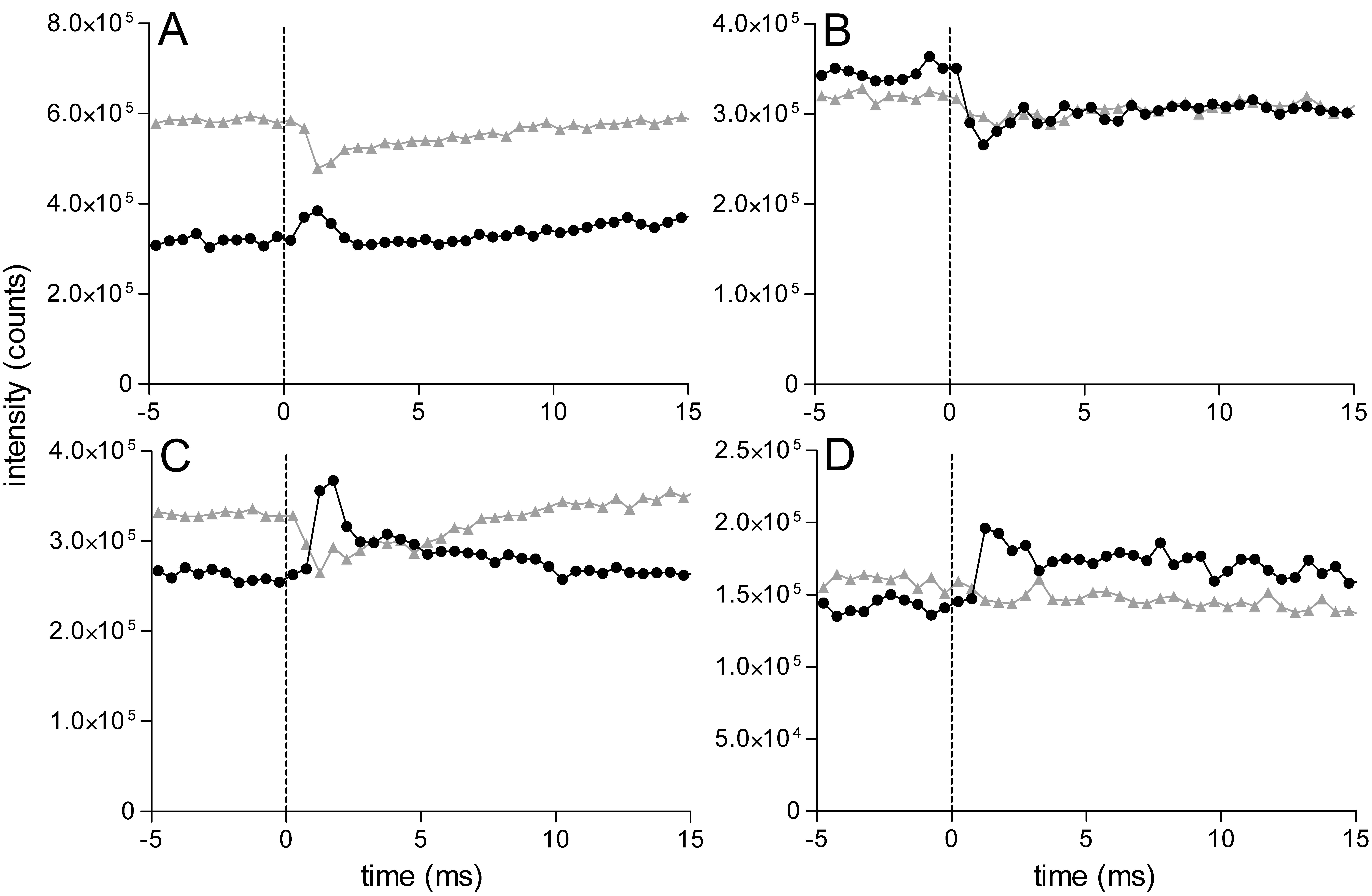


Supplementary Figure 1. Responses of the 111 and 201 reflections to a step stretch of giant waterbug and giant crane fly IFM fibers under fully activating or relaxing conditions. (A) and (B), giant waterbug; (C) and (D), giant crane fly. (A) and (C), at a saturating level of calcium (pCa = 4.0); (B) and (D), relaxing conditions. Black, 111; gray, 201. The numbers of fiber sets used: (A), 18; (B), 10; (C), 8; (D), 7.

Supplementary Movie 1. Fast X-ray diffraction movie recorded from skinned bumblebee IFM fibers, stretch-activated at a saturating level of calcium (pCa = 4.0). Time resolution, 0.5 ms. Reflections of interest are magnified in the boxes, and the simultaneously recorded force is also included (see legend to Fig. 1 for details). Data from 33 sets of parallel-aligned IFM fibers were summed.

Supplementary Movie 2. Responses of the 111 and 201 reflections from bumblebee IFM fibers, activated at various levels of calcium. Note that the responses to stretch become less conspicuous as the calcium level is lowered. For the numbers of fiber sets summed, see legend to Fig. 2.

Supplementary Movie 3. Fast X-ray diffraction movie recorded from skinned bumblebee IFM fibers, step-released at a saturating level of calcium (pCa = 4.0).

Supplementary Movie 4. Fast X-ray diffraction movie recorded from skinned IFM fibers from giant waterbug, stretch-activated at a saturating level of calcium (pCa = 4.0).

Supplementary Movie 5. Fast X-ray diffraction movie recorded from skinned IFM fibers from giant crane fly, stretch-activated at a saturating level of calcium (pCa = 4.0).

Supplementary text

Detailed method for model calculation

The symmetry of myofilaments is based on Tregear et al. (1998). The myosin heads on the myosin filament form a four-start helix, with a basic repeat of 116.1 nm. The monomer pitch of the myosin heads (separation of the myosin head crowns) is 14.5125 nm, and therefore there are 8 crowns per 116.1-nm repeat. The revolution of the helix is 5/16π per crown. Each myosin head is represented as a dot, and its radius from the center of the filament is 10 nm. The basic repeat of the helix of actin filament (the pitch of troponin) is 38.7 nm. A pair of troponins (also represented as dots), 180° apart, exist every 38.7nm level of the actin filament. The radius of troponin from the center of the filament is 5 nm, and the mass of each troponin is half of that of a myosin head. The orientation of troponins is perpendicular to the plane connecting the two neighboring myosin filaments. Both myosin filament backbone and the actin helix per se are regarded as continuous rods, and are therefore excluded from calculation because they contribute only to the equatorial reflections.

A single myosin filament is surrounded by 6 actin filaments, and the troponins on them form a 2-start helix, and the monomer pitch is 38.7/3 nm. The actin target zones, in which actin monomers are favorably oriented for interaction with myosin, is located midway between the two neighboring pairs of troponins, and therefore they also form a two-start helix around a myosin filament.

In calcium-activated fibers, myosin heads close enough to the actin target zone are allowed to interact with actin. The criteria for the closeness are that (1) the myosin head is within azimuthal angles of ± 45°, and that it is axially within 3 nm from the target zone.
